# Correlated states in β-Li_2_IrO_3_ driven by applied magnetic fields

**DOI:** 10.1038/s41467-017-01071-9

**Published:** 2017-10-16

**Authors:** Alejandro Ruiz, Alex Frano, Nicholas P. Breznay, Itamar Kimchi, Toni Helm, Iain Oswald, Julia Y. Chan, R. J. Birgeneau, Zahirul Islam, James G. Analytis

**Affiliations:** 1 Department of Physics, University of California, Berkeley, CA 94720 USA; 2 Materials Sciences Division, Lawrence Berkeley National Laboratory, Berkeley, CA 94720 USA; 3 Advanced Light Source, Lawrence Berkeley National Laboratory, Berkeley, CA 94720 USA; 4 Department of Physics, Massachusetts Institute of Technology, Cambridge, MA 02139 USA; 5 Department of Chemistry, The University of Texas at Dallas, Richardson, TX 75080 USA; 6 Advanced Photon Source, Argonne National Laboratory, Argonne, IL 60439 USA

## Abstract

Magnetic honeycomb iridates are thought to show strongly spin-anisotropic exchange interactions which, when highly frustrated, lead to an exotic state of matter known as the Kitaev quantum spin liquid. However, in all known examples these materials magnetically order at finite temperatures, the scale of which may imply weak frustration. Here we show that the application of a relatively small magnetic field drives the three-dimensional magnet β-Li_2_IrO_3_ from its incommensurate ground state into a quantum correlated paramagnet. Interestingly, this paramagnetic state admixes a zig-zag spin mode analogous to the zig-zag order seen in other Mott-Kitaev compounds. The rapid onset of the field-induced correlated state implies the exchange interactions are delicately balanced, leading to strong frustration and a near degeneracy of different ground states.

## Abstract (web summary)

Materials with a Kitaev spin liquid ground state are sought after as models of quantum phases but candidates so far form either zig-zag or incommensurate magnetic order. Ruiz et al. find a crossover between these states in β-Li_2_IrO_3_ under weak magnetic fields, indicating strongly frustrated spin interactions.

## Introduction

Materials with nearly degenerate ground states are arguably at the center of the most complex and technologically important problems in condensed matter physics. Degeneracies are the reason that strongly correlated materials have rich phase diagrams^1^, can be the origin of topological defects^2^, and are thought to be crucial for the realization of a fault-tolerant quantum computer^3^. One class of such materials are known as quantum spin liquids (QSL). In theory, they are hosts for exotic states of matter, often arising from strong magnetic frustration and characterized by highly degenerate excitations^3, 4^. Honeycomb iridates have ignited interest in this field thanks to their theoretical connection to an exactly soluble QSL discussed by Kitaev^3, 5^.

The Mott–Kitaev iridates crystallize in 2D and 3D structures (the harmonic honeycombs^6^), and all known compounds are found to magnetically order at low temperature in one of two ways: a commensurate “zig-zag” phase (found in α-Na_2_IrO_3_ and α-RuCl_3_^7–9^), and an incommensurate order (found in α, β and γ-Li_2_IrO_3_^6, 10, 11^). This suggests that there are non-Kitaev terms in the Hamiltonian, relieving the frustration and obscuring any low-energy signature of the Kitaev physics^5, 12, 13^. As a result, the presence of Kitaev interactions is often inferred from scattering studies^14, 15^ or from evidence of anomalous dissipative processes in spectroscopic measurements^16, 17^. A central question in these materials is how these different ordered states are connected, and how their strongly correlated nature is evident in their low-energy response functions.

**Fig. 1:**
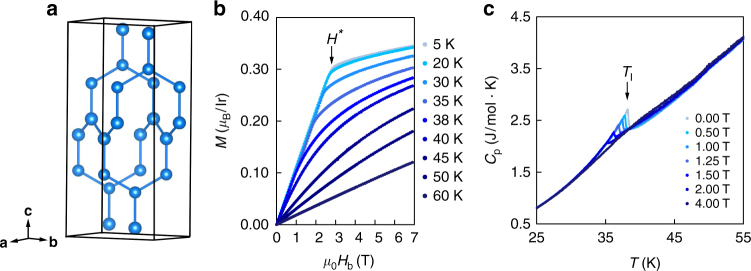
β-Li_2_IrO_3_ structure and thermodynamic properties. **a** 3D projection of a unit cell. The Ir atoms (blue balls) form zig-zag chains stacked along the **c**-axis and directed alternatingly along the **a** ± **b** directions. **b** Magnetization vs. magnetic field applied along the **b**-axis, for temperatures up to 60 K. The low temperature data shows a kink at H*. **c** Heat capacity as a function of temperature for fields up to 4 T. The data shows a triangular anomaly around the transition at *T*_I_

In this work, we find evidence for nearly degenerate broken symmetry states in β-Li_2_IrO_3_, a signature of the underlying magnetic frustration. This compound is the simplest of the 3D harmonic honeycomb structures, composed of interwoven networks of hexagonal chains propagating in the a ± b directions (Fig. 1a). Importantly, Kitaev exchange along the *c*-axis bonds should couple spins pointing in the *b*-axis, making the *b*-axis magnetically special, and thus we focus on the response of the system to an applied field in this direction. We find that in this configuration, a relatively small field can induce a strongly correlated state with zig-zag broken symmetry at the expense of the incommensurate order, providing evidence for strong correlations and a possible familial connection between the low-energy Hamiltonian of different Mott–Kitaev systems.

## Results

### Thermodynamic properties

Single crystals of β-Li_2_IrO_3_ were synthesized using a vapor transport technique as described in Methods. Figure 1b, c show the thermodynamic response of a single crystal with a magnetic field applied along the *b*-axis. In Fig. 1b, we observe an abrupt kink in the field-dependent magnetization at *μ*_0_ H* = 2.8 T, as noted by other authors^10^. This kink occurs at an induced magnetization of M* = 0.31 μ_B_ per Ir, far away from the expected saturation moment ~1 μ_B_ for a *Jeff* = 1/2 ion, and does not show any step-like features characteristic of first-order meta-magnetic transitions. The temperature-dependent heat capacity *C*_*p*_ at various fixed magnetic fields is shown in Fig. 1c. The anomaly at *T*_I=38K_ (*μ*_0_ *H* = 0 T) corresponds to the onset of a known incommensurate magnetic structure with order parameter Ψ_I_ and propagation vector **q** = (0.574, 0, 0)^18^. This highly triangular feature is indicative of a mean-field, second order transition which is strongly suppressed by an applied magnetic field, and completely disappears above H* (Fig. 1c). Assuming H* defines a thermodynamic boundary, it is approximately temperature independent below 25 K.

### Field-dependent resonant magnetic X-ray scattering

**Fig. 2:**
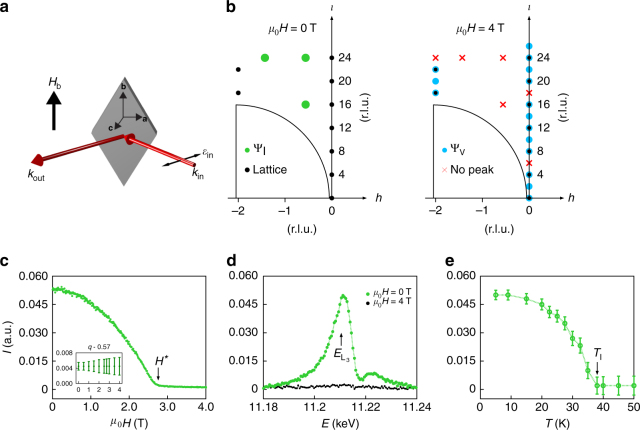
Fate of the incommensurate order **q** = (0.574, 0, 0) under an applied field H_b_. **a** The scattering geometry used during this experiment showing the polarization of the incoming X-ray beam (*π*-polarized) and the direction of the applied magnetic field (along the **b**—axis). **b** The two right panels display all the surveyed positions in reciprocal space at *H* = 0 T (middle panel) and *H* > H* (right panel). Black dots denote structural peaks, blue dots represent the field-induced commensurate magnetic peaks Ψ_V_, green dots represent the incommensurate peaks of Ψ_I_, and the red crosses show absent peaks at *H* > H*. The semicircles represent an inaccessible region below the sample horizon. **c** Field dependence of the scattering intensity at *T* = 5 K showing complete suppression at *μ*_0_ H* = 2.8 T. The inset shows that the **q**—vector remains constant under an applied magnetic field. **d** Energy dependence of the scattering intensity at *T* = 5 K for *μ*_0_ *H* = 0 and 4 T. **e** Temperature dependence at *μ*_0_ *H* = 0 T showing the onset of the order parameter at *T*_I_ = 38 K. The Gaussian fit to the integrated RMXS intensity gives the *χ* ^2^ uncertainty shown by the error bars

To better understand the thermodynamic behavior observed on β-Li_2_IrO_3_, we perform magnetic resonant x-ray scattering (MRXS) at the Ir L_3_-edge (Methods section). This diffraction technique is sensitive to spin and orbital orders, allowing the identification of magnetic ground states in reciprocal space. In Fig. 2a, we illustrate the real space set-up and experimental constraints, while Fig. 2b shows the resulting (*h*, 0, *l*) scattering plane with the accessible reciprocal lattice points at 0 and 4 T. We first study the behavior of the incommensurate state using **Q** = (−0.574, 0, 16). At zero field, the scattering intensity resonates at the Ir L_3_-edge (Fig. 2d) and evolves with temperature like an order parameter Ψ_I_ (Fig. 2e). The response to an applied field at 5 K is shown in Fig. 2c. The peak intensity is reduced with increasing field (completely vanishing at H*), without changing its wavevector **q** (Fig. 2c, inset). In other words, although Ψ_I_ is strongly suppressed in magnitude, its translational symmetry remains rigid.

**Fig. 3:**
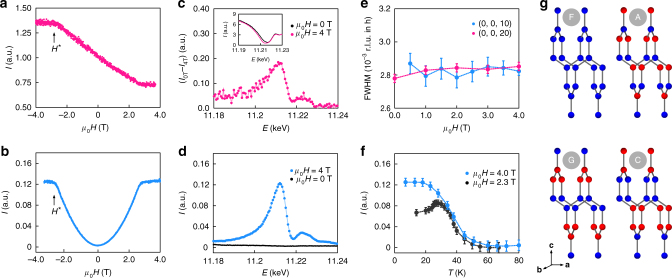
Field, energy and temperature dependence of the commensurate order **q** = (0, 0, 0). Field dependence of the scattering intensity taken at *T* = 5 K and *E* = 11.215 keV around: **a** the structurally allowed (2*m*, 0, 14*n* + 2*m*) peaks (e.g. (0, 0, 20)) which show a linear dependence and, **b** the symmetry disallowed peaks (2*m*, 0, 12*n* ± 2 + 6*m*) (e.g., (0, 0, 10)) which show a quadratic dependence to the applied field. A kink was again observed at *μ*_0_ H* = 2.8 T. The energy dependence for the allowed peaks is shown in the inset **c** with a dip at the absoption edge *E* = 11.215 keV. The main panel **c** shows the difference between the intensity at *μ*_0_ *H* = 0, 4 T which can be attributed to a magnetic contribution. **d** Energy dependence of the magnetic peak (2*m*, 0, 12*n* ± 2 + 6*m*) taken at *μ*_0_ *H* = 0, 4 T. **e** (0, 0, 10) and (0, 0, 20) peaks widths (a lower bound on the correlation length) remain constant under the applied field, suggesting there is no macroscopic phase separation. **f** Temperature dependence of the integrated intensities for the (0, 0, 10) at applied fields above and below H*. Above H* the onset of the FIZZ state is continuous, while below H* this onset is cut off by the incommensurate order. The Gaussian fit to the integrated RMXS intensity gives the *χ* ^2^ uncertainty shown by the error bars in **e**, **f**. **g** Possible basis vectors describing the magnetic order of β-Li_2_IrO_3_, where *F* corresponds to ferromagnetic order, *A* to Néel order, *C* to stripy order and *G* to zig-zag order

We now turn our attention to the properties of this material beyond the phase boundary delimited by H*. Our thermodynamic data indicate a smooth evolution of the entropy-carrying degrees of freedom across H*. While no field-induced changes are observed by MRXS at high symmetry (e.g., *h*, *l* = 1/4, 1/3, 1/2, etc.) nor any other incommensurate positions, we found intensity changes at certain reciprocal *lattice* vectors (blue dots in Fig. 2b). In Fig. 3a, b we plot the field response of the scattering intensity at two kinds of reciprocal space points, one belonging to structurally allowed (2*m*, 0, 4*n* + 2*m*) peaks and the other to structurally forbidden (2*m*, 0, 12*n* ± 2 + 6*m*) peaks, where *n*, *m* are two arbitrary integers. In the former, the response is linear with a negative slope, and shows a kink at H* (Fig. 3a). In the latter case, we find that the peaks have a quadratic dependence on *H*, again with a kink at H* (Fig. 3b). The appearance of structurally forbidden peaks immediately suggests that there is an additional **q** = 0 broken symmetry induced by the applied field.

The energy dependence of these peaks highlights an important contrast in behavior between structurally allowed and forbidden peaks. The former, as shown in Fig. 3c, have a dip at the Ir L_3_-edge due to the resonant absorption of the Ir lattice, while the field-induced change of intensity occurs only near the Ir resonance, suggesting a change in the spin population. Moreover, the structurally forbidden peaks are enhanced at the L_3_-edge in an applied magnetic field (Fig. 3d), suggesting that the **q** = 0 field-induced state is electronic in origin and, as we argue below, most likely magnetic.

In Fig. 3f we illustrate the temperature dependence of the intensity for the field-induced peak with *H* above and below H*. The evolution of the 4 T curve is closely reminiscent of the temperature dependence of a symmetry breaking order parameter, turning on at ~50 K. The 2.3 T curve continuously increases up to *T*_I_, where the incommensurate order turns on. Below *T*_I_, the integrated intensity is reduced, indicating a competition between two coexisting, low temperature states. We assign Ψ_V_ as the parameter describing this field induced broken symmetry which, given that the field dependence of the intensity I_12*n*±2+6*m*_ is quadratic (Fig. 3b), suggests Ψ_V_ is linearly proportional to the scattering form factor. The height of a magnetic Bragg peak can grow by virtue of three things: an increase in correlation length, a canting of the moment that enhances scattering cross section, or an increase in the ordered local moment. In the present case, the intensity of magnetic peaks increases dramatically with *H*, and the peak width remains constant (Fig. 3e). This width is a measure of correlation length and is resolution limited down to the lowest field measured (0.1 T), implying that macroscopic phase separation is highly unlikely. Moreover, a field-enhanced cross-section would imply the existence of a zero-field magnetic order. To see this, note the scattering cross section is proportional to ($\epsilon$_out_ × $\epsilon$_in_)·**m**_**i**_, where $\epsilon$_out(in)_ denotes the polarization state of the scattered (incident) beam, and **m**_**i**_ is a unit vector along the magnetic moment at site *i*. To enhance the cross section, we require a field-induced canting of ordered moments parallel to the term ($\epsilon$_out_ × $\epsilon$_in_), and this can only be achieved for one polarization state at a given incidence angle. Considering we are measuring both the *π* − *σ* and *π* − *π* channels, and see no intensity for all (2*m*, 0, 12*n* ± 2 + 6*m*) peaks at *μ*_0_ *H* = 0 T, we can rule out a zero-field phase with continuous canting of the moments by the field. This implies that our observations are most likely explained by an increasing moment size, with long-range quantum correlations turning on ~50 K, which cannot develop a sizable ordered moment at zero field, presumably due to the system’s intrinsic magnetic frustration.

This behavior differs from archetypical examples of phase transitions in magnetic field such as spin-flops or incommensurate to commensurate transitions^19–25^. These are usually first-order transitions; the former is between an antiferromagnetic state and a spin-polarized state, while the latter will often cause the incommensurate order to soften, shifting toward a commensurate **q** as it is suppressed by the field. In the present case, far from there being a phase transition between one kind of order and another^26^, all broken symmetry states coexist, retaining their intrinsic periodicity as a function of field. The ordered moment is somehow shared between different states, indicating their near degeneracy.

## Discussion

In Fig. 3g, we show the **q** = 0 broken symmetry states that could describe Ψ_V_, denoted using the conventional nomenclature^27^. The symmetries of the state consistent with the primary features of the data above H* are two-fold: *G*-type (zig-zag) broken symmetry, explaining the appearance of the (2*m*, 0, 12 *n*± 2 + 6*m*) peaks and, *F*-type order, explaining the linear dependence of the the allowed structural peaks (2*m*, 0, 4*n* + 2*m*). Importantly, these symmetries are broken by the applied magnetic field itself. First, an induced moment along the applied magnetic field on each ion would transform as an *F*-type object with moments along b (denoted *F*_b_). Second, the effective local susceptibility tensor *χ*_*ij*_ is anisotropic (including orbital *g*_*ij*_-factor as well as correlation effects, see ref. ^6^ for a discussion of *γ*-Li_2_IrO_3_). By symmetry, *χ*_*ij*_ can be decomposed into components parallel and perpendicular to the two honeycomb chain directions along a ± b. This implies that the moments will in general cant along the chains, leading to *G*-type configuration, with moments staggered along *a* (denoted *G*_*a*_). It can be shown (see Supplementary Note 5) that *F*_*b*_ and *G*_*a*_ not only belong to the same irreducible representation as Ψ_I_ (allowing Ψ_V_ to coexist on symmetry grounds), but are in fact energetically favored over other broken symmetry states. Our observations are completely consistent with the Landau theory of second order phase transitions - the combined effect of magnetic field and crystal symmetry is to act as a field on Ψ_V_, so that the observed zig-zag pattern is linearly coupled to magnetic field (leading to an intensity *I*_V_ that is quadratic in field, as seen in Fig. 3b).

**Fig. 4:**
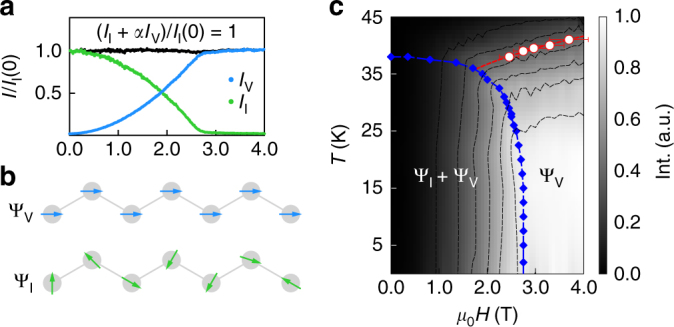
*T*–*H* phase diagram of β-Li_2_IrO_3_ with field along **b**-axis. **a** Ψ_I_ and Ψ_V_ obey a simple sum rule which suggests that spectral weight is exchanged between these parameters as a function of field. **b** Schematic representation of the moment direction in the zero field incommensurate (lower, green arrows) and the field induced zig-zag (upper, blue arrows) order projected on the Ir chains (gray) which propagate in the crystallographic **a** ± **b** directions (see also Fig. 1a). Note the propagation vector of the incommensurate order is not along the chain, but along the **a**-axis. **c** *T* − *H* phase diagram constructed by combining the normalized scattering intensity of a commensurate peak (gray scale contours), with the H* and *T*_I_ extracted from magnetization and heat capacity measurements in Fig. 1 (blue diamonds), and a cusp observed in the field dependence of *C*_p_ (red dots) (determination of the error bars is described in the caption of Supplementary Fig. 3). Note the parameter Ψ_V_ in principle grows as the structure factor and so Ψ_V_ ∝  $\sqrt {{\rm{I}}_{\rm{V}}}$

This scenario explains why there is no sharp thermodynamic anomaly on cooling in the heat capacity at *H* > H* (Fig. 1c): the symmetry breaking associated with Ψ_V_ is already imposed by the applied field. However, the temperature dependence is very similar to that of an order parameter with an onset temperature of ~50 K, even showing signs of competition with the incommensurate state at intermediate fields (Fig. 3f). Moreover, the spin degrees of freedom are clearly frozen out in temperature (at *H* > H*), appearing as a cusp in the heat capacity (Fig. 1c), and as a broad peak in field (see Supplementary Note 3). Plotting the location of this peak on a phase diagram (Fig. 4c), we observe immediately that it maps directly onto a constant contour line of the I_12*n*±2_ amplitude, the experimental measure of Ψ_V_ itself.

The field-induced zig-zag (FIZZ) state may help explain why the magnitude of the induced moment at H* ~ 0.31 μ_B_ per Ir. If we assume that the moment associated with Ψ_V_ is canted entirely along the diagonal bond axes a ± b (which would be equivalent to the zig-zag order seen in α-Na_2_IrO_3_ and α-RuCl_3_) we find a quantitative agreement with the known ordered moment of Ψ_I_. To see this, note the induced magnetic moment at H* implies that the moment along the chains is 0.31/cos(0.2*π*), where 0.2*π* is around half the angle between the diagonal chains. This yields ~0.40 μ_B_ per Ir, accounting for most of the moment in Ψ_I_, measured independently to be ~0.47 μ_B_ per Ir at zero field^27^. The view that only the ordered moment in transferred between the states Ψ_I_ and Ψ_V_ is also consistent with the simple sum rule satisfied by their respective intensities as a function of field (Fig. 4a).

At a fixed field *H* > H*, our data suggest that there is a crossover from a trivial paramagnet at high temperature to a low temperature quantum correlated state. At fixed temperatures *T* < *T*_I_, adding a small magnetic field $({{\mu _0}{H^*}}\sim {T_{\rm I}}{\rm{/}}10{{{{k_{\rm{B}}}}}/{{{\mu _{\rm{B}}}}}} )$ is sufficient to drive the magnetic state of β-Li_2_IrO_3_ from the zero-field incommensurate spiral order Ψ_I_ into the FIZZ state Ψ_V_. Since a small magnetic field drives a transition into the FIZZ spin configuration, it may be natural to interpret this correlated paramagnet as a vestige of a zero-field, zig-zag ground state that is proximate in the thermodynamic phase diagram. This would establish a familial link between the lithium based iridates and α-RuCl_3_/Na_2_IrO_3_^7–9^ by connecting these grounds states through a field-tuned quantum phase transition at H*^28, 29^. In this case, the system’s response at finite field should be understood much like a ferromagnet in an applied field, where the field breaks the same symmetry as the order parameter. As the system is cooled in field, the spins are strongly correlated but they collectively break no new symmetries. We should mention that there is another, yet more exotic interpretation, in which there exists a field-induced spin liquid state, similar to what has been recently suggested as the high-field state of α-RuCl_3_^30^ and γ-Li_2_IrO_3_^31^ (note that in α-RuCl_3_, ${\mu _0}{H^*}\sim {T_{\rm I}}\times{{k_{\rm{B}}}/{\mu _{\rm{B}}}}$). In this scenario, it is only the applied field that directly breaks zig-zag symmetries, and the response of the system is then determined by the temperature/field dependence of the correlations themselves. The onset of spin-correlations will have no thermodynamic discontinuity, as observed in the heat capacity, but may evolve with increasing rigidity in a manner that mimics the appearance of an underlying order parameter, as seen in the I_12*n*+6*m*±2_ intensity. In either case, the ease with which the system can be transitioned from incommensurate to FIZZ states is a striking signature of the delicate balance of interactions in these materials. All that remains is to learn how to tune that balance to achieve a true Kitaev spin liquid.

## Methods

### Single crystal synthesis

Single crystals of β-Li_2_IrO_3_ were synthesized using a vapor transport technique. Ir (99.9% purity, BASF) and Li_2_CO_3_ (99.999% purity, Alfa-Aesar) powders were ground and pelletized in the ratio of 1:1.05. The pellet was reacted at 1050 °C for 12 h, and then cooled down to 850 °C at 2 °C/h to yield single crystals of β-Li_2_IrO_3_. The single crystals are clearly faceted and around 100 × 150 × 300 μm^3^ in size. They are orthorhombic and belong to the *Fddd* space group with lattice parameters *a* = 5.910(3) *Å*, *b* = 8.46(2) *Å* and *c* = 17.85(7) *Å*. The crystal structure is shown in Supplementary Fig. 1a.

### Experimental techniques

Magnetic susceptibility measurements were performed in a 7 T Cryogenic Ltd. SX700 SQUID and Quantum Design MPMS. Heat capacity measurements were conducted on a 16 T Cryogenic Ltd. CFMS micro-chip calorimeter uisng the a.c. method. Resonant X-ray Scattering studies were performed at Beamlime 6-ID-C at the Advanced Photon Source, Argonne National Lab, using a 11.215 keV *π*-polarized x-ray beam and a 4 T split-coil magnet (*σ*-oriented field). Due to the size of the magnet, we were only able to rotate ±3° in the *χ*-direction while keeping the magnetic field parallel to the **b**-axis. Therefore, only the (*h*, 0, *l*) plane was surveyed.

### Data availability

The data that support the findings of this study are available from the corresponding author upon reasonable request.

## Electronic supplementary material

Supplementary Information
