# Supplementary material for: Correlated states in β-Li2IrO3 driven by applied magnetic fields
Source: Nat Commun. 2017 Oct 16;8:961. doi: 10.1038/s41467-017-01071-9 (PMC5643435; doi:10.1038/s41467-017-01071-9)
Supplement: Supplementary file 1 — Supplementary Information [file 41467_2017_1071_MOESM1_ESM.pdf]

Supplementary Note 1: Synthesis and Crystal Structure

Single crystals of $\beta-Li_2IrO_3$ were synthesized using a vapor transport technique. Ir (99.9% purity, BASF) and Li_2CO_3 (99.999 % purity, Alfa-Aesar) powders were grounded and pelletized in air using a molar ratio of 1:1.05. The pellet was placed on a covered alumina crucible and reacted at 1,050° C for 12 hrs, and then cooled down to 850° C at 2° C/hr to yield a powder sample containing single crystals which are clearly faceted and around 105×150×300 μm^3 in size. Room temperature powder and single crystal x-ray diffraction indicated that the high quality crystals were $\beta-Li_2IrO_3$ with an orthorhombic crystal structure and selection rules consistent with the *Fddd* space group.

**Z**	16		
**Space Group:**	*Fddd*		
a,b,c (Å):	5.910(1)	8.462(2)	17.857(6)
α,β,γ (°) :	90°	90°	90°
**Volume (Å^3):**	893.0(5)		

Supplementary Table 1. Structural Parameters of $\beta-Li_2IrO_3$ at 300 K.

Supplementary Fig. 1 shows different projections of the crystal structure of $\beta-Li_2IrO_3$. This 3D structure is locally identical to the 2D honeycomb lattice, $\alpha-Li_2IrO_3$, in which each IrO_6 octahedra shares an edge with three neighbors. The difference arises due to a bonding degeneracy between the edge-sharing octahedra, which results in a three-dimensional network of Ir moments[1].

Supplementary Note 2: Thermodynamic Properties

In this section, we provide further information about the thermodynamic properties of single crystal $\beta-Li_2IrO_3$. Magnetic susceptibility measurements were performed in a 7 T Cryogenic S700X and a Quantum Design MPMS3 SQUID. Specific heat measurements were conducted on a 16 T Cryogenic CFMS using the a.c. calorimetry method, which detects oscillations on the sample's temperature in response to an oscillating heat power[2]. For this, a sample is placed over six thermocouples connected in series under a free standing silicon nitride membrane ∼ 1μm thick. An a.c. current, with frequency ω, is driven through an adjacent resistive heater, resulting on an oscillating power, $P_{ac} = \frac{1}{2}I_o^2R(1+\cos(2\omega t))$. The resulting sample's temperature (V_{ac}) oscillates at frequency 2ω and it is used to calculate the a.c. heat capacity:

$$C_p = \frac{K(T)\cdot P_{ac}}{\omega \cdot V_{ac}} \tag{1}$$

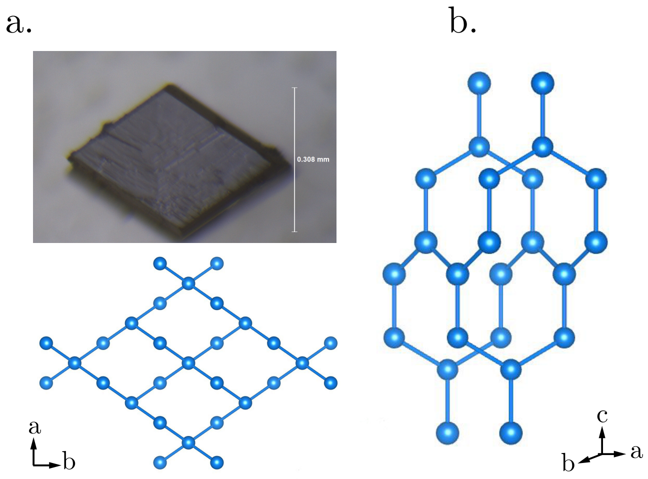

Supplementary Figure 1. (a) Photo of a single crystal of $\beta-Li_2IrO_3$ (upper panel) and projection of the crystal lattice in the **ab**−plane (lower panel). (b) three-dimensional view of a unit cell. The Ir atoms (blue dots) form zigzag chains stacked along the **c** and alternating along the directions **a**±**b**.

These measurements were performed in a low pressure He-4 gas environment (∼ 10 mbar). The optimal frequency used was 20 Hz, necessary to ensure that the thermal link through the membrane and the gas can be ignored, and that the sample is heat homogeneously[3].

Magnetic Anisotropy: Our specific heat and susceptibility measurements showed a clear anomaly at T_I = 38 K, in good agreement with the order state previously reported by Radu Coldea's group (a complex, incommensurate magnetic ground state with non-coplanar and counter-rotating Ir moments that sets in at T_I)[4]. Supplementary Fig. 2 shows the magnetic properties of $\beta-Li_2IrO_3$ with field applied along the principal axes. The response to an applied magnetic field at low temperatures is very anisotropic with χ_a:χ_b:$\chi_c \approx 1:40:10$. A linear Curie-Weiss behavior (Supplementary Eq. 2) was observed for $T > 100$ K, with overall effective moment $\mu_{\rm eff} = 1.81\,\mu_{\rm B}$ per Ir, and Curie-Weiss temperature $\Theta_{\rm CW} = -30$ K.

$$\chi = \frac{\mu_o N_A \mu_{\rm eff}^2 \mu_B^2}{3k_B(T-\Theta_{CW})} \tag{2}$$

The effective moment is very close to the expected value $\mu_{\rm eff} = \sqrt{3}\,\mu_B/{\rm Ir} \sim 1.73\,\mu_B$ per Ir for the ideal J_{eff} = 1/2 moment. The Curie-Weiss temperature is close to the incommensurate transition temperature, $\Theta_{\rm CW} \sim T_I$, as is the case for unfrustrated magnets. However, this value is the result of cancellations between the ferromagnetic and antiferromagnetic interactions, so the frustration parameter, $f = \Theta_{\rm CW}/T_I$, is not a good indication of the degree of frustration in this material. This can be seen when we fit each axis susceptibility independently to a Curie-Weiss model (see Supplementary Table 2). This implies that the interactions along the **b**−axis are weakly fer-

Fitting at 1T along principal axes.			
	$\hat{a}$	$\hat{b}$	$\hat{c}$
μ_{eff} (μ_B/Ir)	1.86 ± 0.1	1.76 ± 0.1	1.99 ± 0.1
θ_{CW} (K)	-94 ± 5	18 ± 5	0 ± 5

Fitting for multiple fields along $\hat{b}$−axis.			
	**1.0T**	**2.0T**	**4.0T**
μ_{eff} (μ_B/Ir)	1.76 ± 0.1	1.73 ± 0.1	1.76 ± 0.1
θ_{CW} (K)	18 ± 5	20 ± 5	18 ± 5

Supplementary Table 2. Curie-Weiss Parameters of $\beta-Li_2IrO_3$ fitted for T > 100 K.

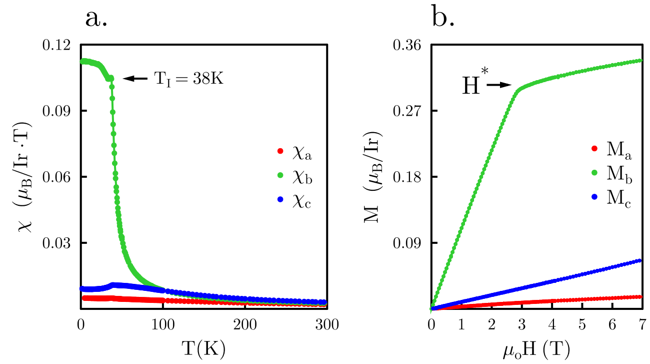

Supplementary Figure 2. **Magnetic properties of single crystal** $\beta-Li_2IrO_3$ **along the three principal axes.** (a) Temperature dependence of the magnetic susceptibility at 1 T. This material orders into an incommensurate magnetic structure at $T_I = 38$ K. Notice the highly anisotropic behavior of this $J_{eff} = 1/2$ system with χ_a:χ_b:$\chi_c \approx 1 : 40 : 10$ (b) Field dependence of magnetization at 5 K. The response of the **a** and **c**−axis is linear with field up to 7 T. Meanwhile the **b**−axis responds linearly up to a kink field H^*.

romagnetic while the **a**−axis interactions have a bigger antiferromagnetic nature. We should also point out that these results vary depending on the temperature range used for the fitting. We have decided to fit our data between 100 − 300 K, since below that, the inverse susceptibility strongly deviates very strongly from a linear behavior and thus, a simple Curie-Weiss model is not applicable.

Supplementary Fig. 2b shows the field dependence of magnetization at $T = 5$ K. Note that there is no sign of hysteresis with field applied along any direction. There is also no difference between field-cooled and zero field-cooled. However, M_a and M_c respond linearly to an increasing magnetic field up to 7 T while M_b has a linear response up to a kink field $\mu_0 H^* \sim 2.8$ T, followed by a gradual increase. The magnetization at H^* is $\sim 0.31\,\mu_B$ per Ir well below the expected μ_B for a fully polarized $J_{eff} = 1/2$ isospin.

b−axis Behavior: Let us turn our attention to the **b**−axis properties of $\beta-Li_2IrO_3$. The edge-sharing IrO_6 octrahedra preserve the essential physics of the Kitaev model where interfering $Ir-O_2-Ir$ exchange paths give rise to orthogonal component of spin and can be described by the Kitaev Hamiltonian[5] which can be relabeled using the crystallographic directions of the 3D orthorhombic honeycomb iridates[1]:

$$\begin{aligned} H_K =& K^\alpha \sum_{\langle ij \rangle}^{\alpha\epsilon(x,y,z)} S_i^\alpha S_j^\alpha \\ =& -K^c \sum_{\langle ij \rangle \epsilon \mathbf{b}} S_i^b S_j^b \\ & -K^h \sum_{\langle ij \rangle \epsilon (\mathbf{a}+\mathbf{c})} S_i^{a+c} S_j^{a+c} \\ & -K^h \sum_{\langle ij \rangle \epsilon (\mathbf{a}-\mathbf{c})} S_i^{a-c} S_j^{a-c} \end{aligned} \quad (3)$$

where $S^{\mathbf{b}}$ and $S^{\mathbf{a}\pm\mathbf{c}} = (S^{\mathbf{a}} \pm S^{\mathbf{c}})/\sqrt{2}$ are the spin operators in a set of orthogonal directions, with **a**, **b**, **c** being unit vectors along the orthorhombic crystal axis. Each $\langle ij \rangle$ bond is defined by the axis perpendicular to its $Ir-O_2-Ir$ plane which lies along one of the directions $\{(\mathbf{a}+\mathbf{c}), (\mathbf{a}-\mathbf{c}), \mathbf{b}\}$. All the nearest neighbor Ir−Ir bonds can be divided into three classes, one for each component of spin: the **b** component from the **c**−axis bonds, and the **a** ± **c** components from the h bonds defining each honeycomb plane. The exchange couplings K^h are constrained by the symmetry of the space group to be the same on the $(\mathbf{a} \pm \mathbf{c})$ bonds, but K^c, the coeficient of S^b coupling, is symmetry-distinct from K^h. Therefore, the **b**−axis is the only crystallographic axis that coincides with an exchange direction in the Kitaev Hamiltonian, making it magnetically special.

Supplementary Fig. 3a shows the magnetic susceptibility as a function of temperature taken at 1, 2, 2.5 & 4 T. For $H < H^*$, the susceptibility is constant, with a maximum value of $\sim 0.11\,\mu_B/\text{Ir}\cdot\text{T}$. However, for $H > H^*$ the magnetic susceptibility monotonically decreases, a behavior typical of ferromagnets. Fitting these data to a Curie-Weiss model yields effective moments $\sim 1.73\,\mu_B/\text{Ir}$ and positive Weiss temperatures indicative of ferromagnetic interactions (Supplementary Table 2). Also notice that χ(T) at $\mu_o H = 4$ T resembles what is expected for a ferromagnetic order parameter under an applied field[6].

The magnetic entropy should be calculated by subtracting the data from a nonmagnetic isostructure. However, since no isostructure currently exists, the heat capacity data at $\mu_o H = 0$ T and 16 T were used to estimate the magnetic entropy change (Supplementary Fig. 3a inset) associated with the transition. At T_I, the value of $\Delta S_m \sim 1.30\,\text{J}/\text{mol}\cdot\text{K}$ which represents $\sim 22.5\%$ of R ln 2, the value expected for the magnetic entropy of a $J_{eff} = 1/2$ moment.

Supplementary Fig. 3b,c illustrate the heat capacity of $\beta-Li_2IrO_3$ as a function of applied field for $T < T_I$ (b)

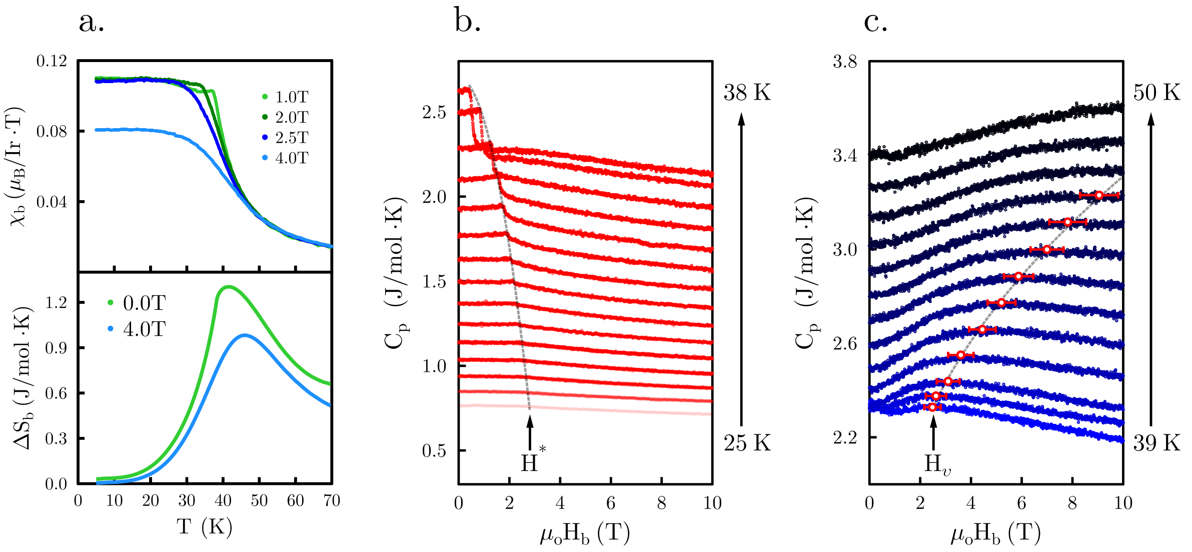

Supplementary Figure 3. **Thermodynamic properties of $\beta-Li_2IrO_3$ with field applied along the $\hat{b}-$axis.** (a) Susceptibility as a function of temperature for μ_oH = 1, 2, 2.5 & 4 T. The lower panel shows the entropy change from at μ_oH = 0 & 4 T with respect to μ_oH = 16 T. (b) Field dependence of the heat capacity for $T < T_I$ and (c) $T > T_I$. A broad hump in the data indicates a crossover into a field−induced phase. A dotted line has been added as a guide to the eye with error bars, of width 2ϵ, determined self consistently using $|y(H_{max}) - y(H_{max} - \epsilon)| = \Delta y$, where Δy is the RMS noise in the measurement.

and $T > T_I$ (c). Supplementary Fig. 3b shows that near T_I the incommensurate phase transition can be easily distinguished as a sharp break of the $C_p(H)$ slope at H^*. This break in slope becomes less notizable as the temperature is lowered, indicating that the phase boundary for the incommensurate phase is strongly vertical which is consistent with our $M(H)$ data (See main text). However, at $T > T_I$, we observe a broad hump in the field dependent heat capacity, the local maximum of which is marked by H_v in Supplementary Fig. 3c. This hump, as well as the reduced ordered moment observed in magnetization measurements and the spreading of the magnetic entropy well above T_I, indicated that the system is highly frustrated and let us to conclude that a new magnetic order was induced by field. We constructed a $T - H$ phase diagram that includes H* (from $M(H)$ measurements), T_I (from $\chi(T)$ measurements), and H_v (from $C_p(H)$ measurements). This phase diagram delineates two low temperature phases: the incommensurate order (INC) for $H < H^*$, and the field−induced zig-zag order (FIZZ) for $H > H^*$. As described below, and in the main text, these two order states coexist below H^*.

Supplementary Note 3: Resonant Elastic X-Ray Scattering

To determine the effect of an applied field along the **b**−axis, we performed resonant x-ray scattering experiments at the Ir−L_3 edge ($E = 11.215$ keV) using a Huber Ψ-diffractometer located in beamline 6ID-C at the Advanced Photon Source - Argonne National Laboratory. The sample used was a clearly faceted single crystal of $\beta-Li_2IrO_3$ about $100 \times 150 \times 150$ μm^3 which was glued onto a copper mount using very low quantities of Stycast 1266 epoxy and, its quality and alignment were checked using the x-ray micro-diffraction facility at the Advanced Light Source - Lawrence Berkeley National Laboratory (beamline 12.3.2). The diffraction experiments were carried out in a reflection geometry, using a π-polarized incident beam $\sim 150 \times 150$ μm^2, with the crystal mounted so that the **b**−axis was parallel to the applied magnetic field. A split-coil magnet, mounted on the cold finger of a closed-cycle He cryostat with three 60° Be windows, provided up to 4 T of continuous magnetic field and sample temperature as low as that of liquid He. A horizontal scattering geometry allowed us to measure both π-π and π-σ channels and the scattering data was collected using a photodiode point detector. Due to the size of the split gap, we were only able to access ± 3.4° in the vertical direction while keeping the magnetic field parallel to the **b**−axis. Therefore, we were only allowed to survey the $(h, 0, l)$ plane.

Linear response to H: The first part of the experiment focused on the field dependence of INC order parameter Ψ_I. For this, we studied the behavior of $(0, 0, 16) + \mathbf{q}$, $(0, 0, 24) - \mathbf{q}$ and $(-2, 0, 24) + \mathbf{q}$ with $\mathbf{q} = (0.57(4), 0, 0)$ and were able to show that $\mu_o H^* \sim 2.8$ T completely suppresses the incommensurate order as illustrated by the supplemetnary fig. 4 b,d (More details on main text).

Since our thermodynamic measurements indicated that a new order could be found above H^*, we continued surveying reciprocal space with $H > H^*$, starting with high symmetry positions (1/4, 1/3, 1/2 etc.) and found that a commensurate order Ψ_V with $\mathbf{q} = (0, 0, 0)$ is enhanced by the applied field and coexist with Ψ_I below

the phase boundary H^* (Figure 4 c,e).

Supplementary fig. 5 shows the field dependence of some selected $(h, 0, l)$ peaks. The top panel shows the response of structurally allowed peaks $(2m, 0, 4n + 2m)$ and the bottom panel shows the emergent, symmetry disallowed peaks $(2m, 0, 12n \pm 2 + 6m)$. Notice that for the structural peaks, the intensity changes linearly with field, while for the purely magnetic peaks, the intensity is quadratic in field. This indicates that in the coexistence region of $\Psi_{\rm I}$ and $\Psi_{\rm V}$, the coupling of the observed ordered states to magnetic field is *linear*. The intensity of a diffraction peak goes as the square of the structure factor so that $I \propto |f_o + \sum_i f_i g(H)|^2$, where f_o is the charge contribution, $g(H)$ is some function describing the response to the magnetic field and f_i is the magnetic structure factor associated with either magnetic order parameter $\Psi_{\rm I}$ or $\Psi_{\rm V}$. For simplicity, let's concentrate on the $(0, 0, l)$ direction. For structurally forbidden peaks, $l = 12n \pm 2$, $f_o = 0$ so that $I_{12n\pm2} \propto |g(H) f_C|^2$. Given $I_{12n\pm2}$ peaks grow quadratically in applied field, this immediately suggests that their response must be linear, $|g(H)| = |H|$. For the structurally allowed peaks, $l = 4n$, $f_o \neq 0$, therefore $I_{4n} \propto f_o^2 + 2 f_o f_V \cdot g(H) + f_V^2 g(H)^2$. The linear dependence of the I_{4n} peaks therefore implies that $g(H) = -H$. By the Landau theory, this would immediately suggest that $\Psi_{\rm V}$ transforms as the magnetic field H.

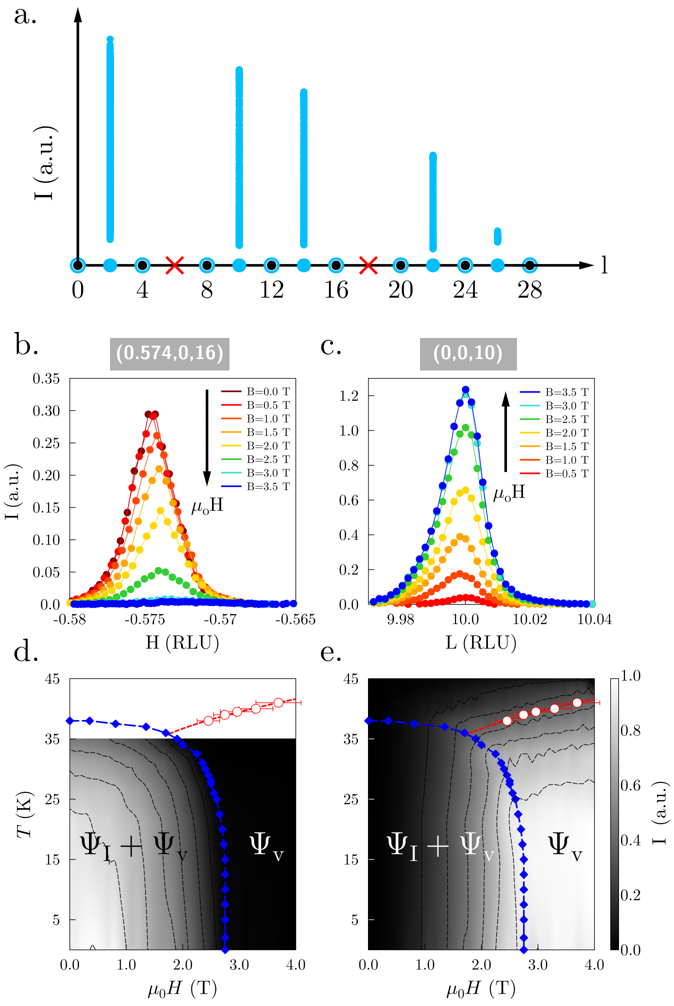

Supplementary Figure 4. Coexistance of incommensurate and zig-zag states. (a) Integrated intensity of the $12n \pm 2$ peaks along (0,0,L). (b-c) Rocking curves for two representatives peaks for the INC and the Zig-zag orders. The INC order intensity is suppresed with field and completely destroyed for $H > H^*$. The zig-zag state is not present at zero field but it starts to form as the field is increased. (d-e) Phase diagram constructed from the thermodynamic measurements superimposed on contour maps of the integrated intensity. Light color indicates a maximum in the intensity while dark means abcense of intensity. Notice that crossover found in the heat capacity, directly maps into a constant contour line of the intensity of the zig-zag order.

Possible ground states for $\Psi_{\rm V}$: We now consider the possible symmetry allowed states for $\Psi_{\rm V}$. There is one magnetic Ir^{4+} site, and four such ions in the $\beta{-}Li_2IrO_3$ primitive unit cell, connected by the symmetry of the $Fddd$ space group. As discussed in previous work[7], any commensurate magnetic order can be represented by a four dimensional vector of the relative phases of the Fourier components at the four magnetic sites in the primitive unit cell. Supplementary Table 3 lists the positions of these ions in the orthorhombic unit cell, with $z = 0.70845(7)$, and the possible basis vectors obtained using the *BasIReps* tool in FULLPROF assuming a magnetic structure with propagation vector $\mathbf{q} = (0, 0, 0)$. According to the language of Ref. 8, F corresponds to ferromagnetic order, A corresponds to Néel order, C to stripy order and G to zig-zag order. The real space configuration of magnetic ions and their relative phases are shown in fig. 3g of main text.

Using these basis vectors, we can derive selection rules for the magnetic scattering using the following form for the structural factor:

$$\mathscr{F}(\mathbf{Q}) = f_S \sum_i \mathbf{M}_i e^{i\mathbf{Q}\cdot r_i} \tag{4}$$

where $\mathbf{Q}$ is the reciprocal lattice vector, $\mathbf{M}_i$ is the ordered moment on the Ir ion at position r_i and the prefactor $f_S = e^{i\pi(h+k)} + e^{i\pi(h+l)} + e^{i\pi(k+l)}$ is the structure factor for the face-centered orthorhombic lattice.

These Bragg peak selection rules are independent of the spin orientation and can be used to distinguish the possible magnetic orders. For clear comparison with experiment, let us focus on the peaks at $k = 0$, i.e. the $(h, 0, l)$ plane. In this plane, the Bragg peaks can be parametrized by two arbitrary integers m, n as follows:

- **FM or structural peaks:** $(2m, 0, 4n + 2m)$, with strong peaks at $(2m, 0, 12n + 6m)$.
- **Stripy peaks:** $(2m, 0, 4n + 2m + 2)$, with strong peaks at $(2m, 0, 12n + 6m + 6)$.
- **Néel peaks:** $(2m, 0, 12n + 6m \pm 4)$.

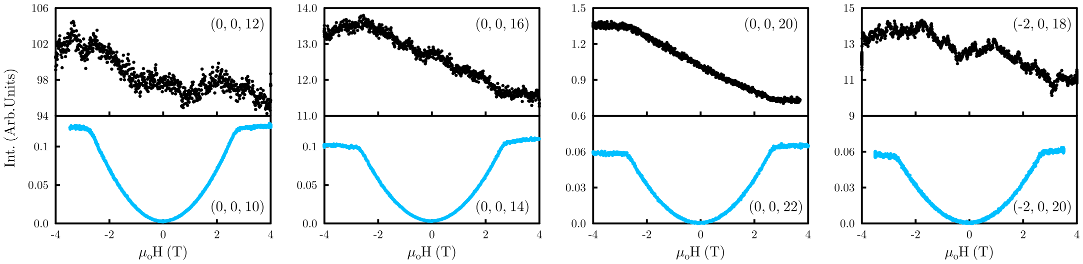

Supplementary Figure 5. Field sweeps at the structural and field-induced peaks. The $(2m, 0, 4n+2m)$ peaks show a linear dependence to an applied field while the $(2m, 0, 4n \pm 2 + 6m)$ respond quadratically, which indicates that the order parameter is linearly coupled to the magnetic field.

Site	Coordinates
1	$(^1/_8,\ ^1/_8,\ z)$
2	$(^1/_8,\ ^5/_8,\ ^3/_4 - z)$
3	$(^3/_8,\ ^3/_8,\ 1 - z)$
4	$(^3/_8,\ ^7/_8,\ ^1/_4 - z)$

Basis Vectors

$$F = \begin{bmatrix} 1 \\ 1 \\ 1 \\ 1 \end{bmatrix} \quad G = \begin{bmatrix} 1 \\ -1 \\ 1 \\ -1 \end{bmatrix} \quad A = \begin{bmatrix} 1 \\ -1 \\ -1 \\ 1 \end{bmatrix} \quad C = \begin{bmatrix} 1 \\ 1 \\ -1 \\ -1 \end{bmatrix}$$

Supplementary Table 3. Ir positions in the orthorombic unit cell and basis vectors for a magnetic structure with propagation vector $\mathbf{q} = (0, 0, 0)$.

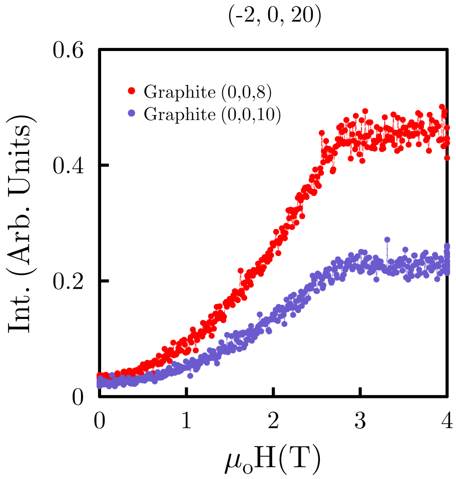

Supplementary Figure 6. **Polarization study of a** $h + l = 12n \pm 2$ **peak.** The polarization measurements were performed using the $(0, 0, 8)$ and $(0, 0, 10)$ directions of a graphite analizer.

- **Zigzag peaks:** $(2m, 0, 12n + 6m \pm 2)$.

If we compare these selection rules to what is experimentally observed at 4 T (Supplementary Fig. 4a), we conclude that a combination of G and F basis vectors is needed to explain our data.

Polarization dependence of Ψ_V: To understand why Ψ_V grows with rising field or decreasing temperature, we note that the cross section for resonant x-ray magnetic scattering at a given $\mathbf{q}$-vector is proportional to $\sum_i e^{\mathbf{i}\mathbf{q}\cdot\mathbf{r}_i}(\boldsymbol{\epsilon}_{out} \times \boldsymbol{\epsilon}_{in}) \cdot \mathbf{m}_i \boldsymbol{\sigma}_i$, where $\boldsymbol{\epsilon}_{out(in)}$ denotes the polarization state of the scattered (incident) beam, $\mathbf{m}_i$ is a unit vector along the magnetic moment at site i, and the sum in i runs over the magnetic unit cell. $\boldsymbol{\sigma}_i$ is a quantity proportional to the local imbalance of magnetic up-down states, and thus proportional to the magnetic moment at site i.

For a π-polarized incoming beam, magnetic resonant scattering occurs in both the π-π and π-σ channels. The product $\boldsymbol{\epsilon}_\pi \times \boldsymbol{\epsilon}_\sigma = \mathbf{k_{in}}$ and $\boldsymbol{\epsilon}_\pi \times \boldsymbol{\epsilon}_\pi = \boldsymbol{\epsilon}_\sigma$ so that the only contribution of the moment to the scattering intensities comes from the parallel projection along the incoming beam, $\mathbf{k_{in}}$, and/or along the normal polarization direction of the beam, $\boldsymbol{\epsilon}_\sigma$. Typically, azimuthal scans are performed to infer the moment's direction by keeping the set-up in the scattering condition and rotating the sample around the scattering wavevector, $\mathbf{Q} = \mathbf{k_{out}} - \mathbf{k_{in}}$. The projection of the moment onto the fixed directions, $\boldsymbol{\epsilon}_\sigma$ and/or $\mathbf{k_{in}}$, varies depending on the azimuthal angle, with maximum scattering for small angle between $\mathbf{m}$ and the fixed directions, and minimum scattering when they are perpendicular. Unfortunately, we were not able to

$\boldsymbol{\epsilon}_{in} \times \boldsymbol{\epsilon}_{out}$	$\mathbf{m} = \mathbf{a}$	$\mathbf{m} = \mathbf{b}$
$\boldsymbol{\pi} \times \boldsymbol{\sigma} = \mathbf{k_{in}}$	$\mathbf{k_{in}} \cdot \mathbf{a} \sim \cos\theta$	$\mathbf{k_{in}} \cdot \mathbf{b} = \mathbf{0}$
$\boldsymbol{\pi} \times \boldsymbol{\pi} = \boldsymbol{\sigma}$	$\boldsymbol{\sigma} \cdot \mathbf{a} = 0$	$\boldsymbol{\sigma} \cdot \mathbf{b} \sim 1$

Supplementary Table 4. The incident beam is π-polarized. The magnetic field is alogn the $\mathbf{b}$-axis, in this geometry, parallel to the σ direction.

perform azimuthal scans to determine the moment's direction and the relative phase between the possible basis vector. However, by probing the polarization of the outcoming beam and by studying the $\mathbf{q}-$dependence along the $(0,0,l)$ direction, we can infer information about their orientation.

Supplementary Fig. 4a shows the $\mathbf{q}-$dependence along the $(0,0,l)$ direction at 4 T. This data can be directly compared with the expected intensity behavior of the π-π and π-σ channels with moments along the $\mathbf{a}$ and $\mathbf{b}-$axis listed on Supplementary Table 4. Since the scattered intensity decreases as the $\mathbf{q}-$vector is increased, we conclude that the moments are mostly along $\mathbf{a}$ and that the major contribution to the intensity is in the π-σ channel.

Polarization analysis were performed using the $(0,0,8)$ and $(0,0,10)$ directions of a graphite crystal with structural factor $F_{(0,0,8)} \sim 4.40$ and $F_{(0,0,10)} \sim 3.24$ and intensity ratio:

$$\frac{|F_{(0,0,8)}|^2}{|F_{(0,0,10)}|^2} \sim 2 \tag{5}$$

which is very similar to that observed on the data (supplementary fig. 6). Since $I = I_{\pi\sigma} + \cos\phi \cdot I_{\pi\pi}$, where ϕ is the polarization angle, and we observed no ϕ dependence in the polarization data (except for what's expected from the intensity ratio), we conclude that most of the intensity comes from the $\pi - \sigma$ channel which agrees with the $\mathbf{q}-$dependence and the energy arguments presented below.

Supplementary Note 4:
Linear coupling of the zigzag order to a uniform magnetic field.

To look for possible coupling between a spatial spin pattern and an external field, such as a spatial modulation of g-factor anisotropy, we first perform a full symmetry analysis of the lattice symmetries.

Supplementary Table 5 show the symmetry transformations of various magnetic configurations under the symmetries of the hyperhoneycomb lattice of $\beta-Li_2IrO_3$, together with time reversal. We find that an external field couples only to a particular configuration, denoted as zig-zag order. The zig-zag order of S^a (S^b) spins couples linearly to a uniform magnetic field along the $\hat{b}$ ($\hat{a}$) axis.

Microscopic mechanisms: Consider the environment of an iridium S = 1/2 site. The local environment of the oxygen octrahedra sets the g-factor anisotropy. The symmetry analysis above shows that it is possible for the material to have a spatially modulated g-factor with off-diagonal terms, akin to that of Sr_2IrO_4. However, here the iridium-oxygen octahedra do not exhibit significant rotations. We therefore expect the magnitude of the site-modulated off-diagonal g^{ab} term to be quite small, less than a few percent.

Once magnetic correlations sample the environment beyond a single Ir site, however, the lattice symmetries immediately come into play. In particular, the local orientation of a zigzag chain produces a preferred local coordinate system for the renormalized magnetic susceptibility. This local coordinate system alternates among sites, in precisely the zigzag pattern.

Symmetry transformations						
		R_a	R_b	R_c	I	T
Uniform field (FM)	S^a	+	−	−	+	−
	S^b	−	+	−	+	−
	S^c	−	−	+	+	−
Stripy	S^a	+	−	−	−	−
	S^b	−	+	−	−	−
	S^c	−	−	+	−	−
Zigzag	S^a	−	+	−	+	−
	S^b	+	−	−	+	−
	S^c	+	+	+	+	−
Néel	S^a	−	+	−	−	−
	S^b	+	−	−	−	−
	S^c	+	+	+	−	−
Noncoplanar spiral		−	0	0	0	−

Supplementary Table 5. Transformation rules for g-factor anisotropies and various magnetic orders under all β-Li_2IrO_3 lattice symmetries. The symmetry generators are: π rotations R around the orthorhombic axes a, b, c, centered at a c-bond midpoint; inversion centers I at the midpoint of d-bonds; and time-reversal T. The space group Fddd also contains glide reflections, which are generated by $R \times I$. The symbols $+, -, 0$ denote that a configuration with a given spin orientation $S^{a,b,c}$ is respectively even, odd, or fully-breaking under the symmetry. The zigzag order of S^a (S^b) spins couples linearly to a uniform magnetic field along the $\hat{b}$ ($\hat{a}$) axis.

One can model this effect as a spatial modulation in a local magnetic susceptibility tensor. The tensor is diagonal in the $\hat{a}, \hat{b}, \hat{c}$ axis, but has an additional off-diagonal component,

$$\chi^{ab} = -\chi^{ba} = (-1)^{\text{zigzag chain}} \tag{6}$$

The sign of this component alternates upon crossing a $\hat{c}$-axis bond, i.e. it alternates between successive zigzag chains.

The effect of this coupling is to produce a zigzag-a (zigzag-b) configuration, together with net $\hat{b}$ ($\hat{a}$) alignment, when a magnetic field is applied along the $\hat{b}$ ($\hat{a}$) axis.

Supplementary Note 5:
Symmetries of the spiral order: Let us consider the spiral order observed in β-Li_2IrO_3. We assume it is incommensurate. The observed spiral has wavevector along a. Its basis vectors all belong to a single irreducible representation Γ_4 at this wavevector, consisting of basis vectors A_x, C_y, F_z, where x, y, z here refer to spin directions along the orthorhombic axes a, b, c respectively. Here F is uniform, and is $\pi/2$ out of phase with the nonuniform basis vectors A and C which are present. For the sites given in the order above, $C = (++--)$ and $A = (+-+-)$.

Now consider its lattice symmetries. As seen in Supplementary Table 5, the operations $I, G_a, G_b, G_c, R_b, R_c$ are

completely broken, in that they each take the spin configuration into a completely different configuration, which remains different even up to an overall spin flip. Thus, the product of each of these symmetry operations with time reversal T also remains broken. However, the remaining operation R_a takes each magnetic moment precisely to its opposite. So its product with time reversal, TR_a, is preserved as a symmetry of the spiral.

This symmetry analysis assumes that translations are fully broken along the spiral wavevector. A commensurate spiral can have a few additional symmetry operations, associated with its very large commensurate unit cell. However, these symmetry operations require fine-tuning of the overall phase of a commensurate order. Lacking any experimental evidence for such phase-locked commensurate ordering, we here focus on the generic case, where the wavevector is incommensurate or, if commensurate, with generic overall phase.

From the analysis above, we see that the spiral ordering preserves only a single space-group symmetry operation: TR_a, the product of time reversal and a rotation by π around the crystallographic a-axis passing through a c-bond midpoint. Aside from the lattice translations along b and c, the spiral order breaks all crystal symmetries except for the single symmetry TR_a.

It is therefore possible for the system to simutaneously develop an order parameter for any order which preserves these symmetries, namely TR_a as well as b, c translations. Together with the a-axis spiral, the following $q = 0$ orders are therefore symmetry allowed:

1. Ferromagnetic alignment along S^b or S^c (FM-b,c).
2. C-Stripy order (spins aligned across z-type i.e. c-type bonds and antialigned elsewhere), with spins again along S^b or S^c (Stripy-b,c).
3. Néel order with spins along S^a (Néel-a).
4. C-Zigzag order (spins anti-aligned across z-type i.e. c-type bonds and aligned elsewhere), with spins again along S^a (Zigzag-a).

For convenience, we only consider the "C" versions of the stripy and zigzag orders, and drop the "C" prefix henceforth.

Energetics of possible vestigial orders: Based on the known information on the Hamiltonian of the zero-field spiral order, which has dominant FM Kitaev exchange, we can estimate the relative energies of these competing vestigial orders. The Néel state is disfavored due to the strong FM exchange along all nearest neighbor bonds. For the stripy pattern, where spins are aligned only along c-bonds, the FM b-axis Kitaev coupling on these c-bonds would favor *Stripy-b*. For the zigzag pattern, where spins are aligned only along x, y-bonds, the FM a, c-axis Kitaev coupling on these bonds would favor *Zigzag-a*.

Finally by observing the known magnetic susceptibility, which is much stronger along b than along a, we note that for the FM patterns, the FM-b is observed to be more easily stabilized than FM-c. The candidate phases with likely lower energy are thus as follows:

1. FM-b;
2. Stripy-b;
3. Zigzag-a.

The Zigzag-a configuration is linearly coupled to FM-b. Applying an external b-field would then be expected to disfavor Stripy-b as well as the more energetically-costly vestigial possibilities, while favoring FM-b together with Zigzag-a.

Quantitative estimate of the Kitaev-Heisenberg exchange: A quantitative estimate of the energetics of the field-induced vestigial order can shed light on the Kitaev-based model for $\beta-Li_2IrO_3$ at zero field. Consider the vestigial order parameter Ψ_V, defined as the magnitude (in units of $\hbar/2$) of the local spin whose magnetic moment orientation is locked to the lattice $b \pm a$ directions. Its energy per site E is given by an expectation value of the $\beta-Li_2IrO_3$ Hamiltonian supplemented by a Zeeman term for the applied field H. Using the K-J-I_c model Hamiltonian for $\beta-Li_2IrO_3$ given in Ref.[8], one finds that the pseudo-dipolar I_c parameter drops out, resulting in the expression:

$$\begin{aligned} E =& -\frac{g\mu_B}{2}(\mathbf{H}\cdot\mathbf{b})\cos\theta\Psi_V \\ &+ \frac{J}{8}(3\cos^2\theta + \sin^2\theta)(\Psi_V)^2 \\ &+ \frac{K}{8}(\cos^2\theta + \sin^2\theta)(\Psi_V)^2 \\ E_V =& -0.047H(T)\Psi_V + (0.292J + 0.125K)(\Psi_V)^2 \end{aligned} \tag{7}$$

With $g = 2$ and $\theta = \tan^{-1} 1/\sqrt{2} \approx 0.2\pi$ corresponding to the $b \pm a$ lattice locking of Ψ_V. Constraints on the magnitude of the ferromagnetic Kitaev ($K < 0$) and antiferromagnetic Heisenberg ($J > 0$) interactions can be derived by comparing E_V to the energy per site of the incommensurate spiral order, estimated via mean-field from $T_I = 38\,K$ to be about $E_I \approx -1.6\,meV$.

Our measurement, showing that a small field $H = 2.8\,T$ is sufficient for destroying the incommensurate order in favor of a saturated vestigial order Ψ_V, implies a high degree of fine tuning between E_V and E_I: taking $\Psi_V = 1$ at $H = 2.8\,T$, we find that the Kitaev and Heisenberg interactions must obey the following constraint,

$$K(\text{meV}) \approx -11.8 - 2.3\,J(\text{meV}) \tag{8}$$

The few-Tesla instability of the incommensurate spiral in favor of the vestigial order thus directly implies that the Kitaev interaction must be significantly larger than the Heisenberg exchange, and indeed must dominate the physical response of the material at both zero and finite applied fields.

Supplementary References

[1] Modic, K. A. *et al.* Realization of a three-dimensional spinanisotropic harmonic honeycomb iridate. *Nature Communications* **5**, 4203 (2014).

[2] Kohama *et al.* AC measurement of heat capacity and magnetocaloric effect for pulsed magnetic fields. *Review of Scientific Instruments* **81**, 104902 (2010).

[3] Riou, O. *et al.* Determination of proper frequency range for accurate heat capacity measurement by AC microcalorimeter. *Superlattices and Microstructures* **35**, 353 (2004).

[4] Biffin, A. *et al.* Unconventional magnetic order on the hyperhoneycomb kitaev lattice in $\beta-Li_2IrO_3$: Full solution via magnetic resonant x-ray diffraction. *Physical Review B* **90**, 205116 (2014).

[5] Jackeli, G. & Khaliullin, G. Mott insulators in the strong spin-orbit coupling limit: From heisenberg to a quantum compass and kitaev models. *Physical Review Letters* **102**, 017205 (2009).

[6] Takayama, T. *et al.* Hyperhoneycomb iridate $\beta-Li_2IrO_3$ as a platform for kitaev magnetism. *Physical Review Letters* **114**, 077202 (2015).

[7] Biffin, A. *et al.* Noncoplanar and Counterrotating Incommensurate Magnetic Order Stabilized by Kitaev Interactions in γ-Li_2IrO_3. *Physical Review Letters* **113**, 197201 (2014).

[8] Kimchi, I., Coldea, R. & Vishwanath, A. Unified theory of spiral magnetism in the harmonic-honeycomb iridates α, β, and $\gamma-Li_2IrO_3$. *Physical Review B* **91**, 245134 (2015).
